# Molecular Basis for Modulation of Metabotropic Glutamate Receptors and Their Drug Actions by Extracellular Ca^2+^

**DOI:** 10.3390/ijms18030672

**Published:** 2017-03-21

**Authors:** Juan Zou, Jason Y. Jiang, Jenny J. Yang

**Affiliations:** Department of Chemistry, Center for Diagnostics and Therapeutics, Georgia State University, Atlanta, GA 30303, USA; jzou2@student.gsu.edu (J.Z.); yusheng0104@gmail.com (J.Y.J.)

**Keywords:** extracellular Ca^2+^, metabotropic glutamate receptor (mGluR), regulation, family C of G-protein coupled receptor (cGPCR)

## Abstract

Metabotropic glutamate receptors (mGluRs) associated with the slow phase of the glutamatergic signaling pathway in neurons of the central nervous system have gained importance as drug targets for chronic neurodegenerative diseases. While extracellular Ca^2+^ was reported to exhibit direct activation and modulation via an allosteric site, the identification of those binding sites was challenged by weak binding. Herein, we review the discovery of extracellular Ca^2+^ in regulation of mGluRs, summarize the recent developments in probing Ca^2+^ binding and its co-regulation of the receptor based on structural and biochemical analysis, and discuss the molecular basis for Ca^2+^ to regulate various classes of drug action as well as its importance as an allosteric modulator in mGluRs.

## 1. Introduction

Ca^2+^, as a first and second messenger, regulates numerous cellular processes through temporal and spatial changes in its concentration and associated changes in the activity of Ca^2+^-receptor/binding proteins. Ca^2+^-binding proteins have Ca^2+^ affinities that vary by 10^6^-fold or more depending on their cellular locations and functions [1,2,3,4,5]. Ca^2+^ interacts with numerous Ca^2+^-binding receptors and Ca^2+^-dependent cell adhesion molecules in the extracellular environment with affinities between 0.1 and 10 mM (*K*_d_), which correspond to the level of the Ca^2+^ concentrations in circulating fluids such as the blood, etc. [6]. Extracellular Ca^2+^ also functions as a first messenger to mediate numerous intracellular functions to trigger diverse cellular processes via family C of G protein-coupled receptors (cGPCR).

Family C GPCRs regulate a number of important physiological functions and are thus intensively pursued as drug targets. Family C GPCRs are characterized by a large amino-terminal domain (extracellular domain, ECD), following a cysteine-rich domain which contains four pairs of disulfide bridges, a transmembrane domain with seven transmembrane sequences (TMS), and an intracellular C-tail segment [7,8]. These receptors function in a dimeric form [8,9,10,11,12]. They play vital roles in sensing vision, taste, and smell; and they couple to the signaling pathway initiated by numerous hormones, neurotransmitters, ions, photons, lipids and designed drugs [13,14]. Family C GPCR from humans is comprised of eight metabotropic glutamate (mGlu1-8) receptors, two heterodimeric-aminobutyric acid B (GABA_B_) receptors, one calcium-sensing receptor (CaSR), three taste (T1R) receptors, one l-amino acid receptor (GPRC6A), and five orphan receptors.

So far, two types of l-Glutamate receptors have been identified, including ionotropic glutamate receptors (iGluRs) and metabotropic glutamate receptors (mGluRs). IGluRs are ligand-gated ion channels, and l-Glu binding leads to subsequent ion influx, causing rapid excitatory postsynaptic current (EPSC). MGluRs are targets of neuron transmitters l-Glu, participating in the modulation of synaptic transmission and neuronal excitability in the central nervous system (CNS). The process of mGluRs converting synaptic l-Glu binding to downstream signaling is relatively slower. In 1987, Kano and Kato et al. demonstrated that activation of mGluRs expressed on Purkinje cells was a cause of long-term depression (LTD) [15]. Four years later, the first subtype of mGluRs was successfully cloned from rat brain by two independent labs, which was named mGluR1 [16,17]. Overexpression of mGluR1 in *Xenopus* oocytes displayed proper function [16,17]. To date, eight different mGluR subtypes have been identified [16,17,18,19,20,21,22,23,24,25] and classified into three groups (group I: mGluR1 and mGluR5; group II: mGluR2 and mGluR3; group III: mGluR4, mGluR6, mGluR7, and mGluR8) based on structural and functional characters [26].

**Figure 1:**
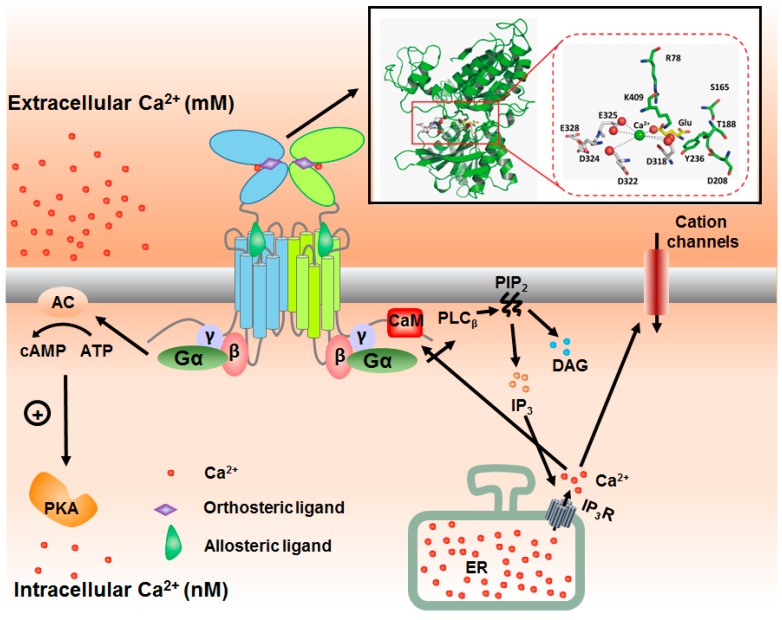
Modulators and signaling pathway of group I mGluRs. Group I mGluRs can be activated by orthosteric modulators independently or be triggered by positive allosteric modulators in the absence of agonists, for example l-Glu or Ca^2+^. Activation of group I mGluRs recruits G-proteins, thereby activating PLC which subsequently decomposes PIP_2_ into DAG and IP_3_. IP_3_ then opens inositol trisphosphate receptor (IP_3_R) on the endoplasmic reticulum (ER) membrane to release Ca^2+^ into cytosol, thus opening the Ca^2+^ channel on cell membrane. At the same time, group I mGluRs also can couple to the cAMP pathway by activating AC which quickly converts ATP to cAMP, thereby activating protein kinase A (PKA).

MGluRs mainly couple to Gα_q_ and Gα_i_ proteins, activating Ca^2+^ and inhibiting cyclic adenosine monophosphate (cAMP) signaling, respectively (Figure 1). Ca^2+^ and cAMP, serving as second messengers, are the census of various signaling pathways within the cells and are involved in multitudes of physiological (learning, memory, anxiety, fear and mood) and pathological processes (Table 1). Group I mGluRs are coupled to Gq. This process in turn activates the cell membrane-bound enzyme phospholipase C (PLC) to decompose phosphatidylinositol 4,5-bisphosphate (PIP_2_) into inositol trisphosphate (IP_3_) and diacylglycerol (DAG), which further modulate protein kinases involved in cascade responses and intracellular Ca^2+^ mobilization, respectively. Group II and III mGluRs are negatively coupled to Gα_i/o_ to inhibit adenylyl cyclase (AC) activity, thus reducing cAMP production [27]. In general, group I mGluRs express around the iGluR core to form an annulus on the surface of post-synapses. Group II mGluRs are mainly distributed on the active zone on pre-synapses to maintain the l-Glu homeostasis in synaptic cleft, although exceptions occur [28]. MGluR6 is related to retinal function, and mutation in mGluR6 leads to night blindness [29,30]. Elevation of l-Glu in the synaptic cleft will cause feedback to pre-synapses.

MGluRs are attractive drug targets for various human diseases. The activity of mGluR1 has been proven to be related to some important physiological and pathological processes, especially neuronal degenerative diseases. Downregulation of mGluR1 was detected in neurons of substantia nigra in Parkinson monkey models, suggesting the important role of mGluR1 in Parkinson disease. Prepulse inhibition (PPI) deficiency usually appears in patients with schizophrenia. Disruption of PPI in mGluR1 knockout mice also suggests that mGluR1 is involved in the process of schizophrenia [31]. In addition, the role of mGluR5 in Fragile X syndrome was supported by the fact that Fragile X symptoms can be reduced by downregulating group I mGluRs. The antagonist of mGluR5, 2-methyl-6-(phenylethynyl)-pyridine (MPEP) is able to suppress the seizure phenotypes [32]. MGluR4 was previously reported as a target to relieve pain. Recent studies suggest that this receptor is also likely to be a therapeutic target for Parkinson’s diseases. An original antagonist *N*-Phenyl-7-(hydroxyimino)cyclopropa[*b*]chromen-1a-carboxamide (PHCCC) of group I mGluRs was reported to enhance the potency of an agonist of mGluR4 l-(+)-2-Amino-4-phosphonobutyric acid (l-AP4). PHCCC is also able to reduce the movement activity in a Parkinsonian rat model [33]. LY2140023, the agonist of mGluR2/3, has been shown to improve both positive and negative symptoms in patients with schizophrenia, and it has entered phase II clinical trials [34]. Recently, overexpression of mGluRs, especially mGluR1 were reported in breast cancer and melanoma, suggesting their involvement in cancer progression [35,36,37,38,39,40,41].

In this review, we will focus on the discovery of extracellular Ca^2+^ in regulation of mGluRs, and recent developments in probing Ca^2+^ binding and its co-regulation of the receptor based on structural and biochemical analysis. The way that Ca^2+^ regulates drug action will also be discussed.

## 2. Extracellular Ca^2+^ and Dynamics in Nervous System

During evolution, cells adapted various ways to exclude high intracellular Ca^2+^, while utilizing its concentration difference in signal transduction. The extracellular Ca^2+^ concentration ([Ca^2+^]_o_) is about 20,000-fold higher than the intracellular Ca^2+^ concentration ([Ca^2+^]_i_). This great difference across the plasma membrane creates the Ca^2+^ gradient from mM (extracellular) to nM (intracellular). Accumulating evidence suggests the crucial importance of extracellular Ca^2+^ and its dynamics in the central nervous system. The dynamic change of Ca^2+^ is localized due to the fact that the synaptic crevices are insulated to serum due to blood–brain barrier. Triggered by presynaptic Ca^2+^ channel activation, the neurotransmitter released from the presynaptic vesicles [42] will induce the synaptic transmission via the postsynaptic receptors. During the sub-millisecond Ca^2+^ influx, Ca^2+^ microdomains and high Ca^2+^ gradients are formed around the presynaptic Ca^2+^ channels. In varying cell types, the local Ca^2+^ concentration ranges from 10 µM to 200 µM for membrane fusion. Additionally, numerous Ca^2+^ sensor proteins on the vesicle membrane surface are activated throughout the neurotransmitter release process [43]. Ca^2+^-dependent inactivation regulates the rapid termination of the Ca^2+^ influx within 1–2 ms [44]. It was estimated that the physiological rest [Ca^2+^]_o_ in the nervous system is about 0.8 to 1.7 mM [45,46,47]. Kubo et al. observed increase of inward Ca^2+^-coupled Cl^−^ current on mGluR1-expressed *Xenopus* oocytes [14,48]. The release of pre-synaptic neurotransmitters was believed to be controlled by Ca^2+^ ions in the synaptic cleft, which in turn modulates the plasticity of post-synapses [49]. In layer 2/3 rat visual cortex, lowering Ca^2+^ in synaptic cleft not only downregulates the exocytosis of neurotransmitter from pre-synapses, but also reduces the post-synaptic efficacy [49]. Furthermore, mGluR1 was proven to modulate the quantal size change of post-synapses. The quantal size was reduced by treatment of either 7-(Hydroxyimino)cyclopropa[*b*]chromen-1a-carboxylate ethyl ester (CPCCOEt) (mGluR1 specific allosteric antagonist), or 2-Amino-3-phosphonopropionic acid (AP3) (group I mGluR specific antagonist) or decreasing Ca^2+^ from 2.5 mM to 1 mM. In contrast, Dihydroxyphenylglycine (DHPG) (the group I mGluR agonist) and Ca^2+^ increase enhanced quantal size [49]. Due to the localized changes and rapid dynamics, accurate measurement of Ca^2+^ concentration and its changes in CNS requires suitable Ca^2+^ probes and biosensor with rapid kinetics and targeting capability [50]. To fill in this gap, our lab developed a fast-kinetic Ca^2+^ reporter CatchER by designing a single Ca^2+^-binding site into enhanced green fluorescent protein [51]. CatchER was able to record the SR luminal Ca^2+^ in flexor digitorum brevis (FDB) muscle fibers during voltage stimulation due to its unprecedented fast off rate [52].

## 3. Integration of Extracellular and Intracellular Ca^2+^ Signaling via mGluRs

Extracellular Ca^2+^ was reported to activate mGluR directly and via an allosteric mechanism [53,54]. In Purkinje cells, mGluR1 is predominant and outnumbers other mGluR subtypes [17]. A [Ca^2+^]_i_ rise in Purkinje cells was detected in responses to extracellular Ca^2+^ exposure, but not in cells from mGluR1 knockout mice [53,54]. However, the response to extracellular Ca^2+^ was restored in Purkinje cells isolated from mGluR1 rescue mice, which express mGluR1 specifically in their Purkinje cells [54]. Applying the mGluR antagonist (*R*,*S*)-α-methyl-4-carboxyphenyl-glycine (MCPG) dramatically decreased this [Ca^2+^]_i_, while blocking the receptor-operated and P-type Ca^2+^ channels by antagonist SKF-96365 and ω-agatoxin IVA did not significantly affect the [Ca^2+^]_i_ responses to extracellular Ca^2+^ [54]. This suggests that the increase of [Ca^2+^]_i_ is not likely to be a result of Ca^2+^ influx through Ca^2+^ channels. In addition to this direct activation, extracellular Ca^2+^ also augmented the cellular responses evoked by l-Glu or its analog. Back in 1998, the [^3^H]-InsP_1_ accumulation evoked by mGluR1 agonist was significantly facilitated by increasing [Ca^2+^]_o_ in baby hamster kidney cells (BHK) [55]. Shigeki et al. analyzed the [Ca^2+^]_i_ increase attributed by mGluR1 activation and found that the initial [Ca^2+^]_i_ increase resulted from Ca^2+^ mobilization from the intracellular Ca^2+^ stores induced by receptor activation, while the sustained phase of [Ca^2+^]_i_ was related to the extracellular Ca^2+^ influx through store-operated Ca^2+^ channels [56]. In CHO-lac-mGluR1 cells, Nash et al. failed to observe any [Ca^2+^]_i_ increase and IP_3_ accumulation [57]. With or without extracellular Ca^2+^, agonist l-Quis induced the similar initial response peak of IP_3_ accumulation, accompanied with [Ca^2+^]_i_ due to Ca^2+^ release from cytosolic Ca^2+^ store [57] (Figure 1). It is worth pointing out that such activation by extracellular Ca^2+^ is largely dependent on the cell types and microenvironment.

Cytosolic Ca^2+^ increases upon extracellular Ca^2+^ activating mGluRs via release of ER Ca^2+^ due to the production of IP_3_. Such increases of [Ca^2+^]_i_ also in turn alter mGluR activity due to changes in the receptor expression on cell surface in several aspects (Figure 1). First, increase of cytosolic Ca^2+^ activates calmodulin (CaM) via cooperative binding of its EF-hand motifs. Ca^2+^/CaM was reported to stabilize the surface expression by interacting with the cytolic tail of several members of mGluRs, including mGluR1, mGluR5 and mGluR7. Interestingly, a protein kinase C (PKC) phosphorylation site (S901) was located within a region of the mGluR5 C terminus which contains a CaM-binding site. Phosphorylation of this site eliminated Ca^2+^/CaM binding, thus reducing surface expression of mGluR5 [58]. On the other hand, preventing S901 from phosphorylation by CaM binding enhances mGluR5 activity [58]. MGluR7 also contains a CaM binding site, which is highly conserved in mGluR4A and mGluR8 [59]. Similarly, phosphorylation of mGluR7 also prevents CaM binding [60]. The accumulating evidence suggests that CaM is the common factor of mGluRs serving as a switch of internalization of the receptors. The role of Ca^2+^/CaM binding in mGluR1 is yet to be elucidated. Second, folding of mGluR and forward-trafficking from the ER to the surface expression are also controlled by the ER Ca^2+^ dynamics. The activity of mGluRs is dependent on the receptor expression on the cell surface. For instance, surface expression of mGluR7 plays an important role in controlling the neuronal plasticity [61]. The decrease of surface mGluR5 by exposure to cocaine leads to loss of endocannabinoid retrograde LTD [62]. MGluRs, like other members of cGPCRs, are folded in ER lumen with the facilitation of chaperones and quality control system. The properly-folded proteins were further modified in Golgi complex, and finally reached the cell membrane. The misfolded receptors are usually uquibinated and protelyzed by proteases. In presence of agonists, the surface receptors will be desensitized and internalized with the assistance of lipid raft and caveolin. Mutants of mGluR1 lacking of caveolin binding motif were demonstrated to attenuate mGluR1 coupled ERK-MAPK signaling pathway [63].

## 4. Key Determinants for Ligand Binding and Activation

There are several important studies revealed the key determinants for extracellular Ca^2+^ sensitivity of mGluRs. Early in 1996, a salmon bifunctional metatropic receptor (sBimR) was cloned from salmon brain, which was highly homological to mGluR1 and the Ca^2+^-sensing receptor [64]. Both extracellular Ca^2+^ and l-Glu in the bath solution could evoke the Ca^2+^ activated chloride current when sBimR was over-expressed in *Xenopus* oocytes. Other polyvalent cations like Gd^3+^ and Mg^2+^ were shown to also induce the Ca^2+^-Cl^−^ current, suggesting that the activation of Ca^2+^-Cl^−^ channels is not solely due to extracellular Ca^2+^ influx. By monitoring the activity of Ca^2+^-Cl^−^ channels in oocytes expressing mGluR1 or mGluR5, Kubo et al. first reported that mGluR1/mGluR5 exhibited sensitivity to extracellular Ca^2+^ [14]. They further demonstrated that group I mGluRs, including mGluR1 and mGluR5, sense Ca^2+^, Mg^2+^, Ba^2+^, Gd^3+^ and other metals. A lower Ca^2+^ activity, however, was also detected in oocytes expressing mGluR3, but not mGluR2. Replacing the entire N-terminal domain of mGluR1 with that of mGluR2 or mGluR3 did not change their sensitivity to l-Glu, but weakened the sensitivity of chimeric mGluR2 to extracellular Ca^2+^ [14]. This finding suggested that the Ca^2+^-sensing capability of mGluRs relies on the extracellular domain. Ser166 in mGluR1 was further suggested to be the key residue contributing to the Ca^2+^-sensitivity of mGluR1 based on mutation studies. Ca^2+^ was shown to induce conformational change of ECD-mGluR3 using single molecule fluorescence resonance energy transfer (FRET), determined between two-tagged SNAP-Alexa647 and CLIP-DY547 with the two protomers of the receptor [65]. Extracellular Ca^2+^ (2 mM) is able to reduce the basal FRET signal on S152D-mGluR2 [65]. Replacing half of the mGluR1 ECD with the corresponding amino acids in mGluR2 ECD altered the agonist selectivity from mGluR1 to a pattern more like mGluR2 [66]. Taken together various functional studies all support the notion that the ECD domain is directly involved in ligand binding.

In 2000, Kunishima et al. reported the first ECD crystal structure of mGluR1 in the presence or absence of its orthosteric ligand l-Glu [67]. Several crystal structures of mGluR ECD were then subsequently determined [68,69]. The Venus flytrap domain (VFT) formed by two globular lobes separated by a cleft or hinge region was conserved in the ECDs of all mGluRs and family C of GPCRs. Both lobes are typical α/β folds where the central parallel β-strands are sandwiched by α-helices. Constitutive dimers formed through the interaction between VFTs from two protomers. A pair of disulfide bonds formed between two protomers was suggested to stabilize the dimer [70,71,72]. Ligand l-Glu was revealed to reside in the cleft /hinge region formed between lobe 1 and lobe 2 of ECD. Interestingly, such a ligand binding site matches the predicted glutamate binding site by Patrick et al. in 1993 using a ECD structure model built based on the sequence homological similarity observed between mGluR1 and bacterial periplasmic binding protein (PBP) [73]. Several putative binding residues (R78, S164, S165, S186 and T188) proposed in the previous studies were shown to directly form hydrogen bonds or water-mediated hydrogen bonds with l-Glu in the determined X-ray structures. Consistently, earlier mutations including S165A and T188A significantly reduced the l-Glu and l-Quis binding affinity. In the open complex form, l-Glu was exclusively coordinated by residues from lobe 1 and residues from lobe 2 were involved in and contributed to additional stabilization force in the closed complex form. Glutamate binding was proposed to change the equilibrium of open and closed conformation and stabilize the closed conformation to activate the receptor [74].

## 5. Seeking for the Molecular Basis of Ca^2+^-Mediated Regulation of mGluRs

The rapid development in structural determination of various forms of mGluRs has provided some important hints for the possible molecular mechanism of calcium regulation. Mg^2+^ was first revealed in crystallization work by Jingami’s group in mGluR1 [67,68]. Mg^2+^ coordinated by L85, L86, I79, and D82 was also reported in a mGluR5 structure (Protein Data Bank (PDB) ID: 3LMK). In 2002, two additional forms bound with l-Glu, Gd^3+^, and (*S*)-α-Methyl-4-carboxyphenylglycine ((s)-MCPG) were reported [68]. These structures revealed three different conformations states upon the bound ligands which were estimated as the activation mechanism of mGluR1. The free form or antagonist-bound form was known as a resting form, also called an open–open form. Upon l-Glu binding, the receptor was stated to be a closed–open form, as lobe 1 and lobe 2 in the same protomer were closed even though two lobe 2 domains were kept open due to the charge repulsion in the interface (E238 and D242). A Gd^3+^ binding site was formed by E238 and D242 from both protomers. Gd^3+^ binding to the negative charge patch between interface of lobe 2 is likely to neutralize electrostatic repulsion and in turn results in toward movement of two lobe 2 domains, forming a close–close form [68]. Whether the lobe 2 domains were closed or open, the metal-induced conformational change is likely to result in a rearrangement of the transmembrane domain with the assistance of a cysteine-rich domain. Mutational studies of the Gd^3+^-binding site confirmed that residue E238 is functionally involved in activation of mGluR1 and modulation of agonist effect on mGluR1. Mutating this residue abolished the Gd^3+^ sensing property while preserving the Ca^2+^- and l-Glu-binding ability of mGluR1 [75]. Consistent with the previous report based on mutagenesis and oocyte current studies [14], mutation S166D abolished Ca^2+^ sensitivity but maintained the Gd^3+^- and l-Glu activity of mGluR1 [75].

## 6. Overcoming the Challenges in Revealing Ca^2+^ Binding Site of mGluRs

To date, more than 30 crystal structures of mGluRs have been determined. Unfortunately, none of them captured Ca^2+^ in the determined ECD structures. There are several major challenges in understanding molecular mechanism of extracellular Ca^2+^ signaling mediated by mGluRs. First, Ca^2+^-binding sites in receptors modulated by high Ca^2+^ (0.05–10 mM) are often invisible even if an X-ray structure could be determined due to the rapid off rates resulting from the low affinity of the Ca^2+^-binding site(s) [76]. Among seven structures of mGluR1 and mGluR5 determined by X-ray crystallography, no Ca^2+^ ions were revealed in any of the determined structures. Further complications arise from Ca^2+^-induced conformational changes and the existence of multiple receptor conformations in equilibrium with each other due to the electrostatic nature of Ca^2+^-binding to charged ligand residues. Second, methods for directly measuring Ca^2+^-binding to mGluR have not yet been established [77,78]. To date, all of the EC_50_ values for Ca^2+^- and amino acid-binding have been determined by indirect functional methods. In addition to its spectroscopic silence and background contamination, the determination of low affinity ligand-binding sites, especially those for Ca^2+^ and amino acids, poses additional challenges. Identifying key determinants contributing to the binding cooperativity and Ca^2+^-induced conformational change are currently largely limited [3,79,80,81,82,83,84]. Third, the large size of the ECD (around 60 KDa) prevents the use of classic high resolution NMR methods for structural studies of ligand-induced conformational changes.

To overcome these challenges and limitations, especially in the visualization of weak Ca^2+^-binding sites in proteins, we have established several innovative methodologies. First, we have developed several computer algorithms for identifying and predicting Ca^2+^-binding sites in proteins based on both structural and sequence information from both apo- and Ca^2+^-loaded forms of X-ray, NMR and modeled structures [85,86,87]. We identified a novel Ca^2+^-binding site in the mGluR1 ECD using a recently developed computational algorithm MUG. This predicted site (comprising D318, E325, D322, and the carboxylate side chain of Glu) is in the hinge region in the ECD of mGluR1, adjacent to the reported Glu-binding site with D318 involved in both Glu- and Ca^2+^-binding. Taking advantage of our established grafting approach by engineering predicted Ca^2+^-binding sites into a scaffold non-Ca^2+^ binding protein, cluster of differentiation (CD2), combined with site-directed mutagenesis, we have successfully verified the intrinsic Ca^2+^-binding capabilities of predicted Ca^2+^-binding sites in the ECD of the mGluR1 [79,81,84,88,89,90,91,92,93,94]. By monitoring Tb^3+^-sensitized luminescence resonance energy transfer (LRET), we are able to probe Ca^2+^ binding affinity upon competition of Tb^3+^-sensitized energy transfer [88]. By performing mutagenesis studies, our lab demonstrated that Ca^2+^ along with l-Glu synergistically activated mGluR1 by binding to a novel Ca^2+^-binding pocket, which partially overlaps the l-Glu orthersteric binding center. Mutations of the l-Glu binding site eliminated l-Glu sensitivity of mGluR1 completely, but only slightly influenced Ca^2+^-coupled signaling. However, Ca^2+^ or Gd^3+^-associated signaling was largely affected by mutations on the Ca^2+^ binding site, while in some cases also suppressing the l-Glu sensitivity of the receptor. Taken together, these data show that it is possible to generate mGluR1 variants responding to either Glu or to Ca^2+^ but not to both. Thus mGluR1 can function as a true Ca^2+^-sensing receptor, since certain mutants, such as S165A and D208I, do not respond to Glu but maintain their Ca^2+^-sensing capacity with only a modest increase in the EC_50_ for [Ca^2+^]_o_ of 2–3 fold. Gd^3+^ is also revealed at the hinge region in the Fourier map, where it shares residues D322 and D324 from the loop that contributes to Ca^2+^ binding [68]. Due to the low resolution of crystal structure (4 Å), the highly flexible loop binding Gd^3+^ in the crystal structure, and the similarity of the binding geometries of Gd^3+^ and Ca^2+^, these two cations probably share, at least in part, the same residues. To address this possibility, the responses to extracellular Gd^3+^ of D318I and E325I were compared with that of the wild type receptor. Dose responses of wild type mGluR1 display a bell-shaped curve consistent with Abe et al.’s data [75,95], while D318I and E325I completely eliminated sensitivity to extracellular Gd^3+^. In view of these findings, we propose a working model of dual activation of mGluR1 by the two physiological activators, extracellular Ca^2+^ and l-Glu, via their overlapping and interacting binding pockets at the hinge region and dimer interface of the lobe 2 of the ECD. Increased concentrations of either Glu or extracellular Ca^2+^ partially activate mGluR1. However, full activation of mGluR1 with maximal sensitivity and a maximal response to Glu requires simultaneous binding of both Glu and Ca^2+^, with D318 playing a key role in the synergy between the two agonists. In this sense, mGluR1 can be viewed as a “coincidence detector”, requiring binding of both ligands for maximal intracellular signaling.

## 7. Extracellular Ca^2+^ Modulates Actions of Orthosteric and Allosteric Drugs

To date, four classes of drugs against mGluRs have been developed. Drugs targeting the endogenous ligand binding pocket were called orthosteric modulators, including agonists and antagonists (Table 2). Usually, orthosteric drugs compete with endogenous ligand for the ligand binding pocket at the hinge region of the ECD. l-Glu analogs, such as l-Quis, have the strongest agonist potency upon mGluR1. They activate mGluRs even in the absence of extracellular Ca^2+^. The highly conserved glutamate binding pocket greatly hindered the development of subtype-specific orthosteric modulators to some extent. In contrast, (s)-MCPG is an antagonist applied to Group I mGluRs, which inhibits l-Glu and Ca^2+^-induced Cl^−^-Ca^2+^ current [14]. Drugs targeting locations other than the orthosteric pocket at the extracellular domain, transmembrane domain, or sometimes C tail are called allosteric modulators (Table 2). Ro 67-4863 is a positive allosteric modulator (PAM) which binds to the transmembrane domain. Ro 67-4863 is unable to activate mGluR1 without Ca^2+^ [96,97]. On the other hand, CPCCOEt, known as a negative allosteric modulator (NAM), inhibits mGluR1 activity also by binding to transmembrane domain [98]. To gain subtype specificity, much effort has been directed to the development of selective allosteric modulator for the treatment of CNS disorders. As a result of intensive investigation, several selective allosteric candidates are very promising for clinical trials (please see an excellent review by Conn [99]). However, the effects of Ca^2+^ binding on the actions of several types of drugs remain unclear [57].

**Figure 2:**
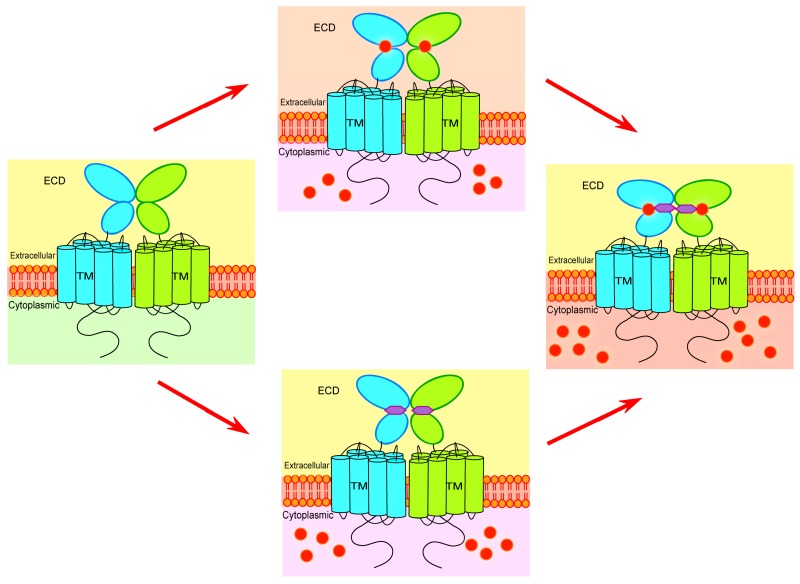
Dual activation mechanism of mGluR1. The schematic diagram shows that l-Glu and extracellular Ca^2+^ synergistically modulate mGluR1-mediated signaling. Elevation of l-Glu or [Ca^2+^]_o_ is able to partially activate mGluR1, while l-Glu and extracellular Ca^2+^ function synergistically to fully activate mGluR1. ECD: extracellular domain; TM: transmembrane.

The synergism of Ca^2+^ and l-Glu to mGluR1 has important applications for the study of other members of cGPCR. (Figure 2). Our lab investigated extracellular Ca^2+^ enhancing the potential of orthosteric agonists (l-Quis) and positive allosteric modulators (Ro 67-4853) and diminishing the inhibitory effects of orthosteric antagonists ((s)-MCPG) and negative allosteric modulators (CPCCOEt) [136]. We firstly found that our predicted Ca^2+^-binding site is adjacent to the orthosteric agonist and antagonist interaction sites and exhibits a good dynamic correlated motion with these sites as assessed by molecular dynamics (MD) simulations. Our studies furthermore demonstrated that [Ca^2+^]_o_ enhances [^3^H]-l-Quis binding to wild type mGluR1 and consistently induces [Ca^2+^]_i_ change . Furthermore, we found that (s)-MCPG efficiently antagonizes both l-Glu- and extracellular Ca^2+^-induced receptor activation at low concentrations, but the increasing concentration of l-Glu or [Ca^2+^]_o_ can overcome this inhibition. Consistent with our studies, the receptor-bound structure with s-MCPG or LY341495 (PDB ID: 3KS9) is shown as a relaxed state [68]. The activation of the receptor was ascribed to the predominance of a close form in dynamic equilibrium although the constitutive activation in mGluR1 is observed upon Homer1b binding to the C terminal tail of the receptor. Such action was further supported by Tateyama et al. using the FRET technique [137]. Two intracellular loop 2 (i2) units were brought closer upon the agonist stimulation (l-Glu and Ca^2+^), while the antagonists increased the space between the loops [137]. In addition, our studies demonstrated that Ro-674853 and CPCCOEt potentiate and inhibit responses to extracellular Ca^2+^, respectively, and extracellular Ca^2+^ increases the potency of Ro-674853 but reduces the inhibition of mGluR1 by CPCCOEt [136]. Therefore, our studies reveal that the binding of extracellular Ca^2+^ to the predicted Ca^2+^-binding site in the ECD of mGluR1 modulates not only glutamate-evoked signaling but also the actions of both orthosteric ligands and allosteric modulators on mGluR1. These studies also open up new avenues for developing allosteric modulators of mGluR function that target specific human diseases.

## 8. Conclusions and Perspective

Recent progress using novel approaches has provided insights for understanding molecular basis of Ca^2+^ modulating group I mGluRs via dual activation. Extracellular Ca^2+^ exhibits strong efforts in modulating several classes of agonists or antagonists to group I mGluR. Since the identified Ca^2+^ binding site residing at hinge region is only conserved in group I mGluRs, this will re-ignite the interest in generating subtype selective orthosteric modulators. Recent structure determinations of CaSR crystal structures also revealed similarities and distinct structural features between mGluRs and CaSR [138,139]. Further investigation and comparison of mGluRs with CaSR activation by Ca^2+^ will enable us to understand the ligand specificity and molecular mechanism of Ca^2+^ activation of family C of GPCR. Further development of the Ca^2+^ sensor to probe Ca^2+^ concentration changes at synapse clefts and ER Ca^2+^ release will also enable us to capture the dynamic role of Ca^2+^ in GPCR-mediated signaling. 

## Figures and Tables

**Table 1:** Key features of metabotropic glutamate receptors (mGluRs). PLC: phospholipase C; MAP: mitogen-activated protein; AC: adenylyl cyclase; cGMP: cyclic guanosine monophosphate.

Group	Receptor	Coupled G Protein	Signaling Pathways	Associated Disease
Group I	mGluR1	Predominantly Gα_q/s_	PLC stimulation, MAP kinase phosphorylation, AC stimulation (some cases)	Schizophrenia, breast cancer, depression, and bipolar disorder
	mGluR5			Schizophrenia, anxiety, chronic pain, Alzheimer’s disease, drug addiction, fragile X syndrome, gastroesophageal reflux disease
Group II	mGluR2	Predominantly Gα_i_	AC inhibition, activation of K^+^ channel, inhibition of Ca^2+^ channel	Anxiety, epilepsy, Parkinson’s disease, depression, addictive disorders, schizophrenia
	mGluR3			
Group III	mGluR4	Predominantly Gα_i_	AC inhibition, activation of K^+^ channel, inhibition of Ca^2+^ channel, stimulation of cGMP (some cases)	Parkinson’s disease
	mGluR6			congenital stationary night blindness
	mGluR7			Schizophrenia, anxiety
	mGluR8			Alzheimer’s disease, Parkinson’s disease

**Table 2:** Orthosteric and allosteric ligands of mGluR1. ECD: extracellular domain.

	**Agonists**			
**Ligand**	**Binding Site**	**Action**	**Potency**	**References**
Quisqualate	ECD	Full agonist	EC_50_: 0.2–3.0 μM	[16,17,100,101,102,103]
ABHx D-I	ECD	Full agonist	EC_50_: 2.0 μM	[104]
3,5-DHPG	ECD	Full agonist	EC_50_: 6.6 μM	[105]
l-glutamate	ECD	Full agonist	EC_50_: 9–13 μM	[16,17,106,107]
(1*S*,3*R*)-ACPD	ECD	Full agonist	EC_50_: 10–80 μM	[102,108,109]
Ibotenate	ECD	Full agonist	EC_50_: 10–100 μM	[110,111,112]
l-CCG-I	ECD	Full agonist	EC_50_: 50 μM	[106]
(*S*)-3HPG	ECD	Partial agonist	EC_50_: 97 μM	[107]
*t*-ADA	ECD	Full agonist	EC_50_: 190 μM	[113]
	**Antagonists**			
**Ligand**	**Binding Site**	**Action**	**Potency**	**References**
AIDA	ECD	Antagonist	IC_50_: 214 μM	[114]
LY341495	ECD	Antagonist	IC_50_: 7.8 μM	[115]
(*S*)-4C3HPG	ECD	Antagonist	IC_50_: 15 μM	[116]
LY367385	ECD	Antagonist	IC_50_: 8.8 μM	[117]
(*S*)-4CPG	ECD	Antagonist	IC_50_: 44–72 μM	[118]
AIDC	ECD	Antagonist	IC_50_: 7.0 μM	[119]
(+)-MCPG	ECD	Antagonist	IC_50_: 3.8 μM	[120]
(*S*)-(+)-CBPG	ECD	Antagonist	IC_50_: 65 μM	[121]
(*S*)-TBPG	ECD	Antagonist	IC_50_: 69 μM	[122]
	**Allosteric Regulators**			
**Ligand**	**Possible Binding Site**	**Action**	**Potency**	**References**
VU-71	7TMD	Positive	EC_50_: 2.4 μM	[96]
Ro 07-11401	7TMD	Positive	EC_50_: 56 nM	[123]
NPS2390	7TMD	Negative	K_i_: 1.4 nM	[124]
R214127	7TMD	Negative	K_D_: 0.9 nM	[124]
JNJ16259685	7TMD	Negative	IC_50_: 3.2 nM	[125]
Ro 67-7476	7TMD	Positive	EC_50_: 174 nM	[126]
Ro 01-6128	7TMD	Positive	EC_50_: 200 nM	[126]
CPCCOEt	7TMD	Negative	IC_50_: 6.6 nM	[98]
Ro 67-4853	7TMD	Positive	EC_50_: 69 nM	[126]
FTIDC	7TMD	Negative	IC_50_: 6 nM	[127]
A841720	7TMD	Negative	IC_50_: 11 nM	[128]
DM-PPP	7TMD	Negative	IC_50_: 15.8 nM	[129,130]
YM298198	7TMD	Negative	IC_50_: 16 nM	[131]
BAY 367620	7TMD	Negative	IC_50_: 160 nM	[132]
EM-TBPC	7TMD	Negative	IC_50_: 15 nM	[133]
CFMMC	7TMD	Negative	IC_50_: 50 nM	[134]
YM-230888	7TMD	Negative	IC_50_: 13 nM	[135]
